# Pattern of Social Interactions after Group Integration: A Possibility to Keep Stallions in Group

**DOI:** 10.1371/journal.pone.0054688

**Published:** 2013-01-30

**Authors:** Sabrina Briefer Freymond, Elodie F. Briefer, Rudolf Von Niederhäusern, Iris Bachmann

**Affiliations:** 1 Agroscope Liebefeld-Posieux Research Station ALP-Haras, Swiss National Stud Farm SNSTF, Les Longs Prés, Avenches, Switzerland; 2 Queen Mary University of London, Biological and Experimental Psychology Group, School of Biological and Chemical Sciences, London, United Kingdom; University of York, United Kingdom

## Abstract

Horses are often kept in individual stables, rather than in outdoor groups, despite such housing system fulfilling many of their welfare needs, such as the access to social partners. Keeping domestic stallions in outdoor groups would mimic bachelor bands that are found in the wild. Unfortunately, the high level of aggression that unfamiliar stallions display when they first *encounter each other* discourages owners from keeping them in groups. However, this level of aggression is likely to be particularly important only during group integration, when the dominance hierarchy is being established, whereas relatively low aggression rates have been observed among stable feral bachelor bands. We investigated the possibility of housing breeding stallions owned by the Swiss National Stud in groups on a large pasture (5 stallions in 2009 and 8 stallions in 2010). We studied the pattern of agonistic, ritual and affiliative interactions after group integration (17–23 days), and the factors influencing these interactions (time after group integration, dominance rank, age or experience of group housing). We found that stallions displayed generally more ritual than agonistic and than affiliative interactions. The frequency of agonistic and ritual interactions decreased quickly within the first three to four days. The frequency of affiliative interactions increased slowly with time before decreasing after 9–14 days. A stable hierarchy could be measured after 2–3 months. The highest-ranking males had less ritual interactions than the lowest-ranking. Males had also less agonistic, ritual and affiliative interactions if they had already been housed in a group the previous year. Therefore, we found that breeding stallions could be housed together on a large pasture, because the frequency of agonistic interactions decreased quickly and remained at a minimal level from the fourth day following group integration. This housing system could potentially increase horse welfare and reduce labour associated with horse management.

## Introduction

Despite being social animals, domestic horses (*Equus caballus*) are very often kept in individual housing systems. This is especially true for expensive horses used for racing and other competitions, because of the potential risks of aggressive interactions such as kicks or bites that could occur when horses are housed together [1]. Stallions used for breeding are also traditionally housed individually, because the high level of aggression that unfamiliar males display towards one another when they first *encounter each other* discourages owners to keep them in groups [1]–[3]. However, individual housing systems can have several disadvantages for horse welfare, and particularly for their mental health, when they are not designed properly (e.g. inducing confinement and preventing social contact [4]–[6]).

Horses housed in individual stables are partially or even totally deprived of physical contact and of activities that are seen under natural conditions, such as locomotion and social behaviours [1], [7]–[9]. Consequently, they display more stress-related behaviours than horses stabled in pairs [10]. They are also likely to develop stereotypies like weaving and cribbing, particularly in stables with minimised contact between neighbouring horses [4]–[6]. Furthermore, a lack of social contact, especially during ontogeny, may predispose horses to impairments in social skills and to an inability to cope with social challenges [2], [11], [12]. Keeping horses in stable groups and in adequate densities could improve welfare, because it would give them access to social interactions, such as affiliative interactions (e.g. play and allogrooming), which have rewarding properties and are indispensable behaviours [2], [9], [13].

Feral stallions (*Equus ferus*) are harem breeders that defend a group of females instead of a particular territory [14]. When they do not have a harem, most stallions form associations known as bachelor bands. These bands contain two to 15 individuals, and are relatively stable over time, although less stable than harem bands. They are composed of yearling or young stallions that have not yet acquired a harem, and are in an intermediate state of development between sexual and social maturity. Bachelor bands can also include older stallions that have lost their harem [15]–[17]. Agonistic and ritualized behaviours like fights, threats, avoidance and submissive behaviours occur among bachelor bands [18], [19]. These aggressive interactions could play an important role in improving skills and physical stamina necessary for stallions to acquire and maintain a harem [16], [19]. However, as in many other species, when they interact, stallions typically display the minimum amount of aggression required by the situation [3]. Therefore, aggression rates are relatively low in natural conditions and encounters rarely escalate into serious fights leading to injuries [18]–[21].

Agonistic interactions, which result in increased distance between two opponents through spontaneous displacement, non-contact or physical aggression, can be prevented by ritualized interactions [3], [22]. Indeed, combat is typically preceded by ritual, threat display and mutual assessment using information about fighting ability from visual, olfactory or acoustical signals [23]. For example, information about familiarity is present in auditory signals such as vocalisations and in olfactory cues, available through behaviour such as dung sniffing [24], [25]. As in many other ungulates (e.g. fallow deer, *Dama dama* [26], [27]; red deer, *Cervus elaphus* [28]), vocalisations also provide information about individuality, body size and dominance status [23], [24], [29]. Ritualized displays, which refer to interactions that do no longer keep their initial function, are common between stallions [17], [19], [22]. These displays typically show a decrease in intensity and duration with time, and seem to facilitate stallions being able to graze side-by-side [19]. They play an important role in establishing and maintaining the hierarchy without involving physical aggression [3], [19].

Housing stallions in outdoor groups is likely to have two main benefits, if enough space is available. First, it could increase horse welfare by allowing them to fully express their natural behaviours including social interactions and locomotion [1], [2], [13]. Second, it could potentially reduce labour required for housing cleaning and exercising horses (H. Besier and I. Bachmann, unpublished data). According to recent reviews on group housing [1], [3], the main reason that prevents owners to keep horses in groups is the potential risk of physical aggression. Several studies have shown that stallions can be kept in stable groups, with few injuries linked to aggressive interactions [18]–[21]. However, physical aggression rates are likely to be particularly high during group integration, when stallions are interacting for the first time and when the dominance hierarchy is being established [3], [30], [31]. Because agonistic encounters and rituals play a role in establishing dominance relationships within a group, we expect their rate and intensity to decrease with time, although not disappear completely, in a stable bachelor group [18]–[21]. More studies are needed to fully evaluate if stallions can be housed in groups, in order to determine aggression levels associated with group integration [1], [3].

In this study, we investigated the possibility of housing breeding stallions owned by the Swiss National Stud in groups on a large pasture. For this purpose, we observed the changes in social interactions over a period of 17–23 days after group integration. We differentiated ritual and affiliative interactions, which do not involve physical aggression, and agonistic interactions, which can potentially involve physical aggression [3], [19]. A rapid decrease in the frequency of agonistic interactions with time would indicate that stallions can be housed in group, because the risk of physical aggression is low after these interactions reach their minimum rate. We also investigated if the final dominance rank, the age or the experience of group housing of stallions affected the frequencies of agonistic, ritual and affiliative interactions during group integration. Finally, we assessed when the dominance hierarchy stabilises.

## Materials and Methods

### Subjects and Management Conditions

The study was carried out at the Swiss National Stud Farm, Avenches, on two groups of Swiss breed stallions (Franches-Montagnes): one group of 5 individuals in 2009 and one group of 8 individuals in 2010. Four individuals were included in both 2009 and 2010 groups (*n = *9 stallions in total). These stallions were 8–19 years old and had been kept at the Swiss National Stud for 5–16 years. They were used for breeding and for driving. They had all, but one, been regularly hitched next to each other for driving. Before the study, they had been housed on several occasions in adjacent stables, but they had never been in a group. Therefore, all the stallions used in this study were familiar with each other, but had no experience of group housing.

Because prior exposure can reduce aggression between horses during physical encounters [30], the stallions were housed for 14 days next to each other in indoor individual stables (9 m^2^) separated by partitions with a rail at the top half, allowing them to interact. They could therefore hear, see, smell and partially touch each other. When housed in individual stables, in 2009, the stallions were individually put in a pasture for two hours per day. In 2010, they were exercised four by four in a horse walker for one hour per day. They were given feed mix three times a day and were provided with hay two times a day and straw.

Stallions were then moved together to an outdoor pasture (4 hectares) for six months. Horseshoes were removed before group integration in order to minimize the risks of injuries. In pasture, hay was distributed during winter according to horses’ needs. Pasture fences and horse health was checked daily. Dung was cleaned once a week. In case of high summer heats, an insecticide was applied daily or weekly as required. Six wood shelters (5 of 9 m^2^ and one of 15 m^2^) with wide stabilised entrances and whose ground was covered with straw were available for horses within the pasture. The pasture did not contain any closed spaces. Food was well distributed to ensure that every horse could feed easily without being threatened or kicked by other horses. Finally, the group was housed in a pasture away from mares and other horses. After the study, stallions were put back in their previous individual stables and used for breeding.

### Group Integration Procedure

Following a preliminary experiment in 2008, in which four stallions were successfully integrated together, we repeated the same procedure. In July 2009 and 2010, the stallions were handled individually on a halter and brought to the pasture. The persons handling the stallions walked once around the pasture and then released all the stallions at the same time. Ten people holding driving whips were present and ready to intervene in case of serious fight. The vet team of the Swiss National Stud Farm was present during the integration and checked horse health on a daily basis throughout the experiment.

### Observations

Social interactions were scored daily either at 09∶00 h–11∶00 h, 13∶00 h–15∶00 h and 17∶00 h–18∶00 h, or at 07∶00 h–09∶00 h, 11∶00 h- 13∶00 h and 15∶00 h–17∶00 h from the first hour to the 557^th^ hour (23 days) after group integration in 2009 and to the 413^th^ hour (17 days) after group integration in 2010. Because the frequency of interactions was considerably higher during the first two days after integration, these data were analysed later from videos filmed by two experimenters. Data for the rest of the study were scored by direct observation by two experimenters. All data were collected from an observatory post, from which the whole pasture (i.e. all horses at all time) was visible. In total, the behaviour of each stallion was scored during 109 hr in 2009 and 87 hr of observation in 2010.

We scored the frequency of the following social interactions (defined in Table 1) continuously using the behaviour sampling rule, i.e. by observing the whole group and scoring every interaction with details of which individuals were involved: agonistic interactions; ritual/investigative interactions and affiliative interactions. Agonistic interactions were defined as non-contact or contact interactions that resulted in increased distance between two stallions (e.g. chase, push and kick; Table 1). Ritual/investigative interactions (thereafter “ritual interactions”) were defined as non-contact interactions between two stallions used to assess each other’s social status without fighting (i.e. faecal pile display, sniff and sniff and squeal; Table 1). Affiliative interactions (i.e. non agonistic and non ritual) included allogrooming (or mutual grooming) and play (Table 1 [3], [17], [19], [22], [32], [33]). Interactions were analysed as frequencies per hour per horse.

**Table 1:** List and description of the interactions scored after group integration.

Behaviour	Description
***Agonistic interactions***	
*Chase*	Chasing another horse, ears laid back with the neck extended and exposing the teeth.
*Push*	Pushing with the head the neck, shoulder, chest, body or rump of another stallion.
*Kick threat*	Raising a hind leg in the direction of another stallion, but without touching him, ears laid back.
*Kick*	Kicking another horse with one or the two hindlegs.
*Strike*	A rapid motion of one or both forelegs in the anterior direction.
*Bite threat*	Neck stretched, teeth exposed and ears laid back, pretending to bite without touching the other horse.
*Bite*	Biting another horse, lips retracted, ears laid back with the muzzle muscles tensed.
*Nip*	Biting another horse, but without the ears laid back and with the mouth less widely open than during a real bite.
*Mount*	Mounting another stallion, similarly as during copulation.
*Lunge*	One stallion rears with the forelegs in the direction of another horse, ears laid back.
*Circling*	Two stallions circle each other head-to-tail, trying to nip or bite each other’s body parts.
*Kneeling*	Two stallions circle each other and drop on one or both of their knees.
*Fleeing*	Avoiding, retreating from another horse by walking, trotting or galloping, usually with ears laid back.
*Following*	Walking behind another horse, head low without any attempt to attack or bite. This behaviour was scored only in 2010.
***Ritual/investigative interactions***	
*Sniff and squeal*	Two stallions sniff each other’s muzzle, body parts or genitals, with the neck arched and produce a squeal.
*Faecal pile display*	Sequence of behaviours associated with defecation onto a faecal pile. Typically, two or more stallions defecate on a faecal pile, turn around, sniff the pile and scratch the ground with a foreleg.
*Sniffing*	Olfactory investigation of another horse’s muzzle, body parts or genitals, with the neck arched, but without squealing like during *sniff and squeal.*
***Affiliative interactions***	
*Play*	Two stallions nip each others’ body parts, without their ears laid back, while moving or not.
*Mutual grooming*	Two stallions groom each others’ neck, back or rump by gentle nipping, nuzzling, or rubbing while standing head-to-tail.

The categories of interactions that were included in the analyses are shown in bold and the behaviours scored are in italic. A short description of the behaviours is included when needed (see also [3], [17], [19], [22], [32], [33], [36]).

### Dominance Relationships

We tested dominance relationships once a month, during three months after group integration using pair feeding tests [34], [35]. These tests consisted in placing a bucket of carrots between each possible pair of stallions. Videos of the tests were analysed and the stallion that chased the other one away to eat in the bucket was considered as dominant and the other horse as subordinate. Because dominance hierarchies in horses are generally linear, particularly in the case of small groups such as in our study [3], [36], the dominance index for a given male was then calculated according to [37] as follows: [(number of horses that this male dominates – number of horses that this male is dominated by + group size + 1) / 2]. The male with the lowest index value in each year was assigned the rank of 1 and all other males were ranked accordingly. Therefore, higher values of rank indicate higher-ranking males. We used the final dominance rank measured after three months to investigate the effect of the hierarchy on the frequency of interactions.

### Statistical Analyses

#### Social interactions in group

We used generalized linear mixed model (GLMM) fit by the Laplace approximation (lmer function in R [38]) to investigate the effects of the time after group integration, the age and dominance rank of stallions, the number of matings they performed and their experience of group housing on the frequency of social interactions.

We first tested if, independently of the time after integration, stallions favoured one category of interactions over the others (antagonist, ritual, affiliative). To this aim, we carried out a GLMM including the frequency of interactions (frequency per hour per horse; 109 frequencies per hour per horse in 2009 and 87 frequencies per hour per horse in 2010; mean±SE = 99.6±3.0) as a dependant variable, the time after integration (1–557 hours) as a control factor, and the category of interaction (antagonist, ritual, affiliative) as a fixed effect. We also included as random effects the year of observation (2009 or 2010), to account for between year differences, and the individual identity of horses, to account for repeated measurements of the same individual within and between years. This model was fit with residual maximum likelihood estimation (REML). We carried out more GLMMs including the same fixed and random factors as described above for two-by-two comparisons and we applied a Bonferroni correction at α = 0.017 (0.05/3).

We then used a model selection procedure based on the Akaike’s information criterion adjusted for small sample size (AIC_C_) to identify the factors (time after group integration, dominance rank, age or experience) that best explained each of the three categories of interactions (antagonist, ritual and affiliative; frequency per hour per horse [39]). All models were fit with maximum likelihood estimation (ML). We formulated one set of candidate models for each of the three interaction categories (Table 2). Within each set of models, the first model consisted of the random effects only (null model; model 0), which were the year of observation and horse identity. In the next model, we included the time after group integration (1–557 hours) as a fixed effect (model 1). Because this factor was highly significant (Table 2), it was included as a control factor in all the other models. In addition, we included as a fixed effect the final dominance rank after three months (1–5 in 2009 and 1–8 in 2010; model 2), the age of the stallions (8–19 years old; model 3), or their experience of group housing (*i.e.* if they had been housed in group already the year before: coded as 1 for 2010 horses that were in group in 2009 and 0 for the others; model 4).

**Table 2:** Models fit to investigate the effects of the time after group integration (“Hours”), the age (“Age”) and the dominance rank (“Rank”) of stallions, and their experience of group housing (“Experience”) on the frequency of interactions (agonistic, ritual or affiliative).

Response variable	Model	Fixed effect(s)	AICc	ΔAIC_C_	*wi*	ER	Model comparison	*X^2^* (df)	*P*
**Agonistic**	0	None	630.75	590.95					
	1	log(Hours)	42.82	3.02	0.15	4.53	1 vs 0	589.95(1)	**<0.0001**
	2	log(Hours) + Rank	44.51	4.70	0.06	10.51	2 vs 1	0.34(1)	0.56
	3	log(Hours) + log(Age)	43.46	3.66	0.11	6.22	3 vs 1	1.39(1)	0.24
	**4**	**log(Hours) + Experience**	**39.80**	**0.00**	**0.68**	**1.00**	**4 vs 1**	**5.04(1)**	**0.025**
**Ritual**	0	None	651.31	700.02					
	1	log(Hours)	−42.63	6.07	0.04	20.80	1 vs 0	695.96(1)	**<0.0001**
	**2**	**log(Hours) + Rank^2^**	**−48.70**	**0.00**	**0.81**	**1.00**	**2 vs 1**	**10.11(2)**	**0.006**
	3	log(Hours) + Age^2^	−42.53	6.18	0.04	21.93	3 vs 1	3.94(2)	0.14
	4	log(Hours) + Experience	−44.81	3.89	0.12	7.00	4 vs 1	4.20(1)	**0.040**
**Affiliative**	0	None	−3153.89	40.69					
	1	Hours^2^	−3191.06	3.51	0.10	5.80	1 vs 0	41.21(2)	**<0.0001**
	2	Hours^2^ + Rank^2^	−3192.89	1.68	0.26	2.32	2 vs 1	5.88(2)	0.053
	3	Hours^2^ + Age^2^	−3189.62	4.95	0.05	11.09	3 vs 1	2.61(2)	0.27
	**4**	**Hours^2^ + Experience**	**−3194.58**	**0.00**	**0.59**	**1.00**	**4 vs 1**	**5.54(1)**	**0.019**

*Note.* The response variable (category of interaction) and fixed effect(s) included in the models are indicated. The fit of the models is assessed by Akaike’s information criterion corrected for small sample sizes (AIC_C_): the lowest value for a given response variable (i.e. set of models) indicates the best fit (in bold). ΔAIC_C_ gives the difference in AIC_C_ between each model and the best model. The Akaike’s weights (*wi*) assess the relative support that a given model has from the data, compared to other candidate models in the set. The evidence ratio (ER) is the ratio between the Akaike’s weight of the best model and that of a competing one. Results of the likelihood-ratio tests (*χ^2^* and *p*) used to compare the various models (“Model comparison”) and to assess statistical significance of the factors are indicated (significant results are in bold). Fixed effects: “Hours” indicates a linear term, “log(Hours)” a log term and “Hours^2^” a quadratic term (indicating that both linear and quadratic terms were included in the model).

Within each set of model, when the difference between the AIC_C_ values of two models (ΔAIC_C_) is less than 2 units, both models have support and can be considered competitive. Models with ΔAIC_C_ ranging from 3 to 7 have considerably less support by the data, models with ΔAIC_C_>10 are poorly supported, and ΔAIC_C_>20 have no empirical support [39], [40]. Akaike weights (*wi*) indicate the probability that a particular model has more or less support from the data among those included in the set of candidate models [39]. For each model, we also calculated the evidence ratio, defined as the ratio between the Akaike weight of the best model and the Akaike weights of the competing model, to determine to what extent it was better than another. Additionally, we used the likelihood-ratio tests (LRT) to compare models within a given set and to assess statistical significance of the factors, by comparing the model with and without the factor included (Table 2).

We fit fixed effects as linear, quadratic or log terms based on the lowest AIC_C_ value (Table 2). All categories of interactions were log-transformed and fit with a Gaussian family distribution and identity link function. Q-Q plots and scatterplots of the residuals of the dependent variables were inspected visually to ensure their normal distribution.

#### Stability of the hierarchy

To measure the stability of the hierarchy over time, we calculated, for each year, Kendall rank correlations (“Kendall’s tau”) between the dominance ranks of the stallions measured after one month and their ranks after two months, and between their ranks measured after two month and their ranks after three months (Table 3).

**Table 3:** Dominance hierarchy after one, two and three months (final rank) following group integration.

Year	Stallion	Dominance rank after		
		One month	Two months	Three months (final)
**2009**	Havane	3	4	5
	Lordon	3	4	4
	Naguar	3	3	3
	Nico	2	2	2
	Valentino	1	1	1
**2010**	Havane	7	6	8
	Naguar	6	5	7
	Nico	4	4	6
	Lordon	5	4	5
	Laura	4	4	4
	Nestor	3	3	3
	Van Gogh	2	2	2
	Commodore	1	1	1

The hierarchy appeared stable after two (2010) to three months (2009) following group integration. Higher dominance ranks indicate higher-ranking males.

We carried out statistical analyses using R v.2.9.0 [41]. All means are given with standard errors (SEs).

### Ethics

Keeping horses in outdoor groups is a housing system allowed by welfare regulations. All animal work was conducted in accordance with the relevant local guidelines (Swiss law on animal protection and welfare). No experiment with animals has been performed in our study. The health of stallions was checked on a regular basis by veterinarians of the Swiss National Stud Farm. None of the stallions had to be removed from the group because of injuries caused by social interactions.

## Results

### Social Interactions in Group

The time after group integration (1–557 hours; GLMM: log term, *z = *−34.16, *p*<0.0001) and the interaction category (antagonist, ritual, affiliative; GLMM: *z = *−31.19, *p*<0.0001) had an effect on the frequency of interactions. Further tests showed stallions displayed, independently of the time after integration, more ritual interactions (4.60±0.20 interactions per hour) than agonistic interactions (3.17±0.22 interactions per hour) and than affiliative interactions (0.30±0.02 interactions per hour; *n = *1241 frequencies for each interaction category; GLMM: ritual versus agonistic, *z = *10.92, *p*<0.0001; ritual versus affiliative, *z = *34.15, *p*<0.0001; agonistic versus affiliative, *z = *−21.48, *p*<0.0001; Bonferroni correction: α = 0.017).

**Figure 1:**
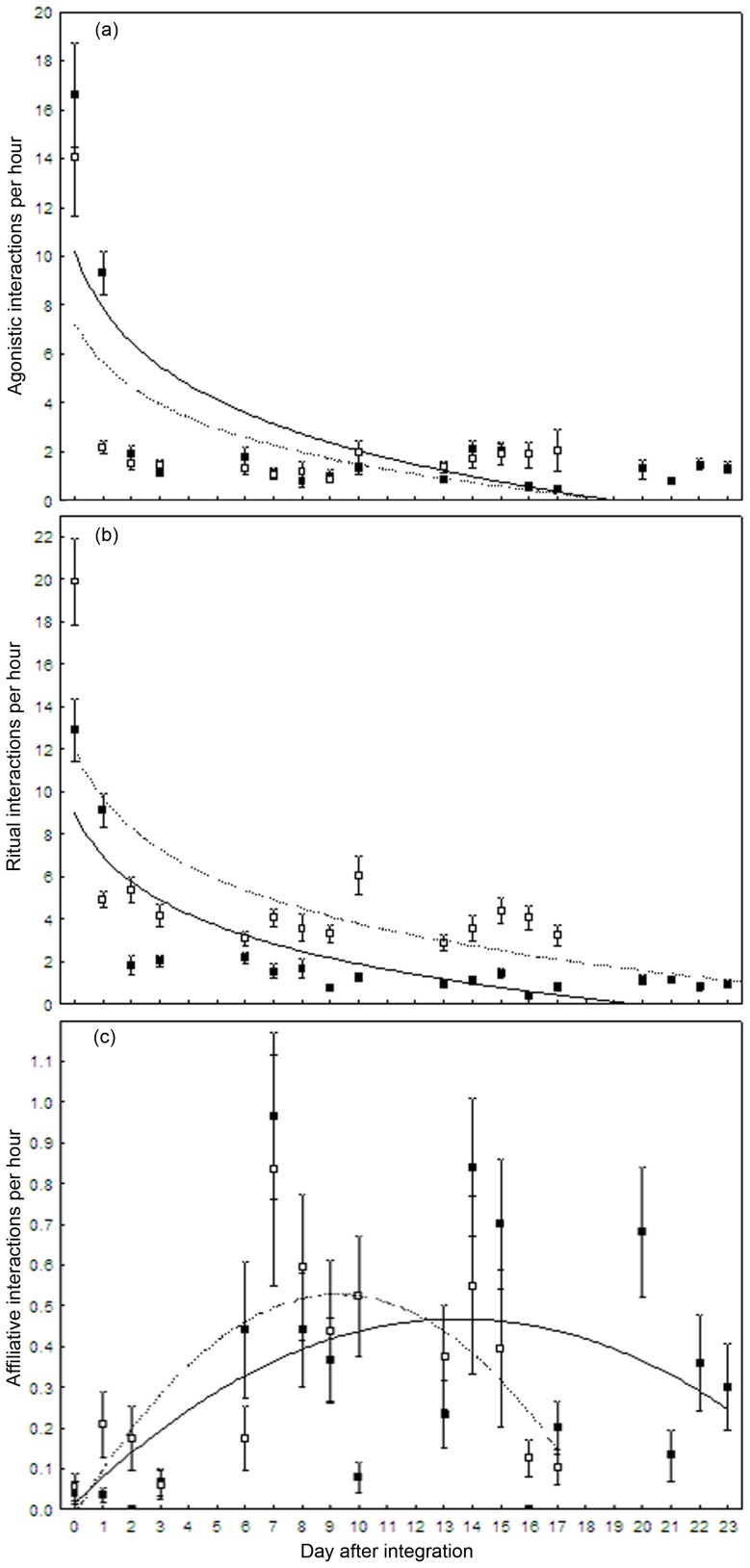
Changes with time in the frequency of social interactions after group integration. Frequency of interactions per hour (mean±SE per day; agonistic (a), ritual (b) and affiliative (b) interactions) as a function of time (days) in 2009 (black square) and in 2010 (empty squares). The best fit (log or quadratic) is indicated with a solid line for 2009 and dashed line for 2010 data.

**Figure 2:**
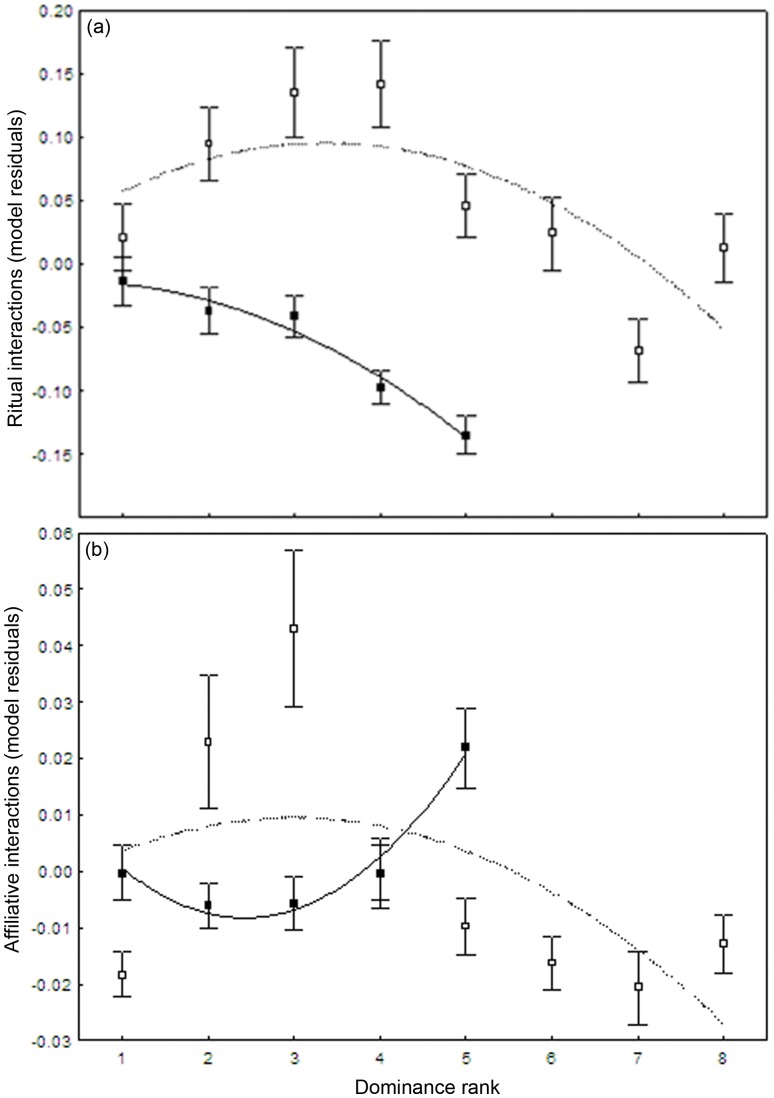
Relationship between the frequency of ritual (a) and affiliative (b) interactions per hour (model residuals controlled for the effect of the time after integration) and the dominance rank of stallions in 2009 (black square) and 2010 (empty squares; mean±SE per rank). The best fit (quadratic) is indicated with a solid line for 2009 and dashed line for 2010 data. Residuals represented stallions that had more interactions than predicted by the time after integration. Higher dominance ranks indicate higher-ranking individuals.

The model selection procedure based on AIC_C_ showed that the time after group integrations explained the largest amount of variation in the frequency of all categories of interactions (Table 2). Agonistic and ritual interactions decreased quickly with time, whereas affiliative interactions increased during the first days and decreased later on (Fig. 1). The experience of group housing was also a good predictor of all categories of interactions, with males having fewer interactions when experienced. The dominance rank of stallions was a good predictor of the frequency of ritual interactions (Fig. 2a).

Adding the time after integration to the null model significantly improved the models explaining all categories of interactions (model 1; Table 2). This parameter explained a large amount of variation in the data, particularly for agonistic and ritual interactions (ΔAIC_C_ between model 0 and model 1: agonistic interactions, 587.93; ritual interactions, 693.95; affiliative interactions, 37.17). The frequency of agonistic and ritual interactions decreased rapidly after group integration (3–4 first days) and was maintained at its lowest values for the rest of the study both in 2009 (mean interactions per hour before day 4: agonistic, 8.92±0.88; ritual, 7.84±0.64; *n = *160 frequencies; after day 4: agonistic, 1.22±0.07; ritual, 1.17±0.06; *n = *385 frequencies) and in 2010 (mean interactions per hour before day 4: agonistic, 5.28±0.77; ritual, 9.07±0.74; *n = *256 frequencies; after day 4: agonistic, 1.56±0.15; ritual, 3.84±0.18; *n = *440 frequencies; Fig. 1a and b). The frequency of affiliative interactions increased from day 0 to day 14 in 2009 and from day 0 to 9 in 2010 and decreased afterwards (mean±SE: 2009, 0.30±0.03; *n = *545 frequencies; 2010, 0.30±0.04; *n = *969 frequencies; Fig. 1c).

Adding the dominance rank (model 2) to model 1 significantly improved the model explaining ritual interactions, but not agonistic interactions (not significant) and affiliative interactions (trend only; Table 2). In 2010, the frequency of ritual interactions increased from ranks 1 to 4, and then decreased from ranks 5 to 8, whereas it mainly decreased with rank in 2009, with higher-ranking individuals having less ritual interactions (Fig. 2a). Adding the dominance rank tended to improved the model explaining the frequency of affiliative interactions, although this was only a trend (likelihood-ratio test: *X* ^2^ = 5.88, *p = *0.053). In 2009, the frequency of affiliative interactions was higher in the top-ranking stallion (rank 5) compared to lower-ranking ones, whereas the opposite seemed to occur in 2010, with affiliative interactions being highest in males with rank 2 and 3 and decreasing in higher ranking stallions (Fig. 2b).

Adding the experience of group housing significantly improved model 1 for all categories of interactions (Table 2). In 2010, horses with no experience of group housing had more agonistic interactions (model residuals controlled for the effect of time after integration: −0.025±0.015), more ritual interactions (0.098±0.016) and more affiliative interactions (0.012±0.005; *n = *4 horses and 1241 frequencies) than horses that were already in group in 2009 (agonistic interactions = −0.050±0.014; ritual interactions = 0.003±0.013; affiliative interactions = −0.015±0.003; *n = *4 horses and 1241 frequencies).

As a result, the model that best explained the variation in the frequency of agonistic and affiliative interactions was the model including both the time after integration and the experience of group housing (model 4; Table 2). This model had 68% chance to be the best model within the set of models explaining agonistic interactions, and 59% chance to be the best model within the set of models explaining affiliative interactions. The model that best explained the variation in the frequency of ritual interactions was the model including both the time after integration and the dominance rank of stallions (model 2; Table 2). This model had 81% chance to be the best model within the set of models explaining ritual interactions. Within the set of models explaining affiliative interactions, the model including the time after integration and the dominance rank of stallions (model 2) was a close competitor of model 4 (ΔAICc<2; Table 2). This model had 26% chance to be the best model. All the other models had considerably less support by the data (ΔAICc>3). To summarize, the best model explaining the frequency of agonistic and affiliative interactions included the time after integration and the experience of group housing, and the best model explaining the frequency of ritual interactions included the time after integration and the dominance rank of stallions.

### Stability of the Hierarchy

A stable hierarchy was established and could be measured after two (2010) to three months (2009; Table 3). In 2009, the hierarchy was stable after 3 months (correlation between dominance ranks measured after 2 and 3 months; Kendall’s tau = 1.00, *n = *5 horses, *p = *0.027), but not after 2 months (correlation between dominance measured ranks after 1 and 2 months; Kendall’s tau = 0.84, *n = *5 horses, *p* = 0.096). In 2010, the hierarchy was already stable after 2 months (Kendall’s tau = 0.96, *n = *8 horses, *p = *0.002), and was still stable after 3 months (Kendall’s tau = 0.95, *n = *8 horses, *p = *0.002; Table 3).

## Discussion

Unlike individual housing systems, group housing allows horses to fully express their natural behaviours [2], [3], [9], [13]. The main reason that prevents owners to keep horses in groups is the potential risk of physical aggression, or a lack of suitable grazing land. The risk of physical aggression is likely to be particularly high during group integration, when the dominance hierarchy is being established. In this study, we investigated social interactions occurring after stallions had been integrated into a new group, in order to assess the potential risks of aggressive interactions such as kicks or bites between horses. We showed that stallions displayed generally more ritual than agonistic and than affiliative interactions. Agonistic and ritual interactions decreased within a few days following group integration (three to four days), while affiliative interactions increased slowly with time before decreasing later on. A stable hierarchy was established between group members after two to three months. The males at the top of this hierarchy after three months had less ritual interactions than the lower-ranking ones during the observation period (17 to 23 days after group integration). Males had also less agonistic, ritual and affiliative interactions if they had already been housed in group the previous year, suggesting an effect of social experience on interactions. Therefore, under the specific tested conditions, stallions can be kept in groups, because agonistic interactions are maintained at a minimum rate after the first few days following group integration, which corresponds to the rate observed among wild bachelor groups (1.4 interactions per hour in our study versus 1.5 in natural populations of Przewalski’s horse, *Equus ferus przewalskii* [20]). We therefore encourage horse breeders with extensive pasture land to keep stallions in stable groups and in adequate densities [3], [19]–[21], particularly for those that are not used for breeding the whole year around. This could potentially improve horse welfare and reduce labour associated with horse management (H. Besier and I. Bachmann, unpublished data).

### Pattern of Social Interactions after Group Integration

We found that the time after group integration explained a large amount of the variance in the data. Agonistic and ritual interactions decreased quickly within the first three to four days after integration. These changes were very similar between the two groups studied in 2009 and 2010. After that, the frequency of agonistic interactions that we measured (1.40 h^−1^ per horse) was similar to the frequency measured by Christensen et al. [20] in a bachelor group of Przewalski’s horses (1.46 h^−1^ per horse; *n = *13 stallions), but higher than the frequency measured in Bourjade et al. [17] (0.2 h^−1^ per horse; *n = *9 Przewalski’s stallions) or in a smaller bachelor group (0.76 h^−1^ per horse; *n = *4 Przewalski’s stallions [42]). In contrast, affiliative interactions increased slowly with time and then decreased after 9–14 days.

Social interactions play an important role in the establishment and maintenance of hierarchies. Within a social group, a stable hierarchy functions to regulate aggression and thus reduce the number of serious fights [43]. When two males *encounter each other*, they perform a ritual that allows them to assess each other’s fighting abilities using information contained in visual, olfactory or acoustical signals, without having to fight [23]. Accordingly, in our study, stallions had generally more ritual than agonistic interactions, thus preventing real fights [36]. These mutual assessments are effective alternatives to real aggression, but can escalate into serious fights over resources of any kind, when the degree of asymmetry in fighting abilities between the two individuals is low, or if there is an ambiguous hierarchy [3], [21], [43]. In contrast, the increase in the frequency of affiliative interactions at the beginning of the study indicated that social bonds were being established. In horses, typical affiliative behaviours are play, allogrooming and anti-parallel standing rest [9], [32]. Play behaviour is particularly displayed in groups of males or mixed gender groups, compared to female groups [33]. The main function of affiliative relationships is to reduce social tension between group members and therefore, to increase group cohesion [2], [13]. We suggest that the following decrease in affiliative interaction observed after 9–14 days in our study could be due to the fact that the frequency of affiliative interactions required to establish social bonds is higher than the frequency required to maintain these bonds. Therefore, once relationships have been established, the frequency of affiliative interactions could decrease [2].

### Factors Affecting Social Interactions

The time after group integration was the main predictor of the frequency of interactions. However, other factors, such as the dominance rank of stallions and their experience of group housing also played a role. Ritual interactions were lower in higher-ranking stallions compared to lower-ranking ones. Stallions experienced in group housing had less agonistic, ritual and affiliative interactions than other stallions.

Our results showed that the frequency of ritual interactions, but not agonistic interactions, was influenced by the hierarchy. Similarly, in Przewalski’s horse natural populations, lower-ranking stallions have been shown to engage more often in rituals than higher-ranking ones, which could indicate that they have a tendency for compromising rather than fighting [17]. High-ranking stallions win more fights, but do not to display higher rates of physical aggression than other males [3], [17]. This suggests that the dominance rank of high-ranking males is rarely challenged. Threats, olfactory cues and vocal cues may suffice to maintain their dominance rank. Tilson et al. [18] found that conflict for rank was limited to the three lower-ranking Przewalski’s stallions, within a group of eight bachelors. Because mutual assessments are more frequent when the degree of asymmetry in fighting abilities between two individuals is low [43], these results suggest that the degree of asymmetry in fighting abilities is generally lower at the bottom of the hierarchy, or in our 2010 group, within the stallions that were ranked 2–4.

Affiliative interactions tended to be affected by dominance rank, with higher ranking males displaying more affiliative interactions in 2009, and less affiliative interactions in 2010, than low-ranking ones (trend, *p = *0.053). In other studies, affiliative interactions have been shown to be more often initiated by dominant individuals, as we found in 2009 (e.g. [44], [45]). Low-ranking individuals might rarely initiate affiliative interactions with higher-ranking individuals, because of the elevated risks of provoking an agonistic interactions [44], [46]. Therefore, dominant individuals are expected to contribute more than subordinate to affiliative relationships, because they can choose whom to bond with, whereas subordinates cannot [47]. In our 2010 group however, which was a larger group than in 2009, the relationship between rank and frequency of affiliative interactions was less clear. The frequency increased from ranks 1 to 3, followed by a decrease in high-ranking stallions. Our observations were collected during the first 17–23 days following group integration, while the hierarchy was being established. Indeed, our measures of the stability of the hierarchy revealed that it was stable after three months in 2009 and two months in 2010. We suggest that in large groups, while the hierarchy is being established, dominant individuals could have less affiliative interactions than their subordinates while trying to maintain their rank in the hierarchy.

Our results show that stallions had also less agonistic, ritual and affiliative interactions if they had already been housed in group the previous year. These results could be linked to an increase in familiarity between stallions. Indeed, Hartmann et al. [30] showed that pre-exposing unfamiliar horses by placing them in adjacent stables reduces both aggressive and non-aggressive interactions when they physically meet for the first time. However, the stallions used in our study had been regularly hitched next to each other for driving and had been housed at several occasions in adjacent stables before the first group integration. Therefore, all the stallions used in this study were already familiar when we first housed them in a group. An alternative explanation would be that these results are linked to stallions’ experience of group living. Previously singly stabled stallions have been shown to display more aggressive interactions (e.g. bite threat), but also more affiliative interactions (allogrooming and play), than previously group housed ones [2]. These results could be due to a build-up of motivation during the period when horses are kept individually, suggesting that stallions are sensitive to social deprivation and that individual housing has long-term negative effects on social behaviour [2]. Furthermore, horses might need to acquire social competences in order to behave appropriately in group [3], [11], [48]. The proportion of “inappropriate” threats directed towards more dominant individuals decreases with age [44], indicating an important role of experience on social skills [3]. Horses that have been living in group have more refined social skills and are less aggressive towards other horses and even towards humans during training [2], [12], [49], [50]. Therefore, these results suggest that the stronger the social experience of horses that are integrated in a group is, the lower the frequency of agonistic interactions would be. Further experiments, in which stallions are unfamiliar to each other before group integration, could help to disentangle the effects of familiarity and experience of group living on the frequency of interactions.

By definition, a natural behaviour is important for animal welfare if performing this behaviour improves the animal’s physical or mental health [51]. A behaviour is considered as an “ethological need” if it is performed by all individuals, is self-rewarding, has a rebound effect and if chronic stress, which can lead to abnormal behaviour, is triggered when the performance of this behaviour is prevented [52]. In horses, allogroming, and to a lesser extend play, have been identified as ethological need because they meet all criteria [2], [9]. A lack of social contacts triggers stress-related behaviours and stereotypies in horses. Social interactions should therefore be considered as crucial for welfare [4]–[6]. Many individual stables afford horses no opportunity to interact with neighbours. However, where possible, stables should be designed to allow adjacent neighbours to physically interact through, for example, partitions with vertical bars at the top half.

### Conclusions

Housing horses in groups fulfils many of their welfare needs, including the access to social partners and the establishment of a social structure [1], [9]. Such system could potentially increase horse welfare and reduce labour associated with horse management. In this study, we showed that stallions can be housed in groups under specific conditions, because agonistic interactions, which are potentially linked to physical aggression, decrease and are kept at a minimum rate after only three to four days following group integration.
